# Open-source platform to benchmark fingerprints for ligand-based virtual screening

**DOI:** 10.1186/1758-2946-5-26

**Published:** 2013-05-30

**Authors:** Sereina Riniker, Gregory A Landrum

**Affiliations:** 1 Novartis Institutes for BioMedical Research, Basel, Switzerland

**Keywords:** Virtual screening, Benchmark, Similarity, Fingerprints

## Abstract

Similarity-search methods using molecular fingerprints are an important tool for ligand-based virtual screening. A huge variety of fingerprints exist and their performance, usually assessed in retrospective benchmarking studies using data sets with known actives and known or assumed inactives, depends largely on the validation data sets used and the similarity measure used. Comparing new methods to existing ones in any systematic way is rather difficult due to the lack of standard data sets and evaluation procedures. Here, we present a standard platform for the benchmarking of 2D fingerprints. The open-source platform contains all source code, structural data for the actives and inactives used (drawn from three publicly available collections of data sets), and lists of randomly selected query molecules to be used for statistically valid comparisons of methods. This allows the exact reproduction and comparison of results for future studies. The results for 12 standard fingerprints together with two simple baseline fingerprints assessed by seven evaluation methods are shown together with the correlations between methods. High correlations were found between the 12 fingerprints and a careful statistical analysis showed that only the two baseline fingerprints were different from the others in a statistically significant way. High correlations were also found between six of the seven evaluation methods, indicating that despite their seeming differences, many of these methods are similar to each other.

## Background

The concept of molecular similarity is often used in the context of ligand-based virtual screening (VS) to use known actives to find new molecules to test [1]. Molecular similarity is also used for applications such as the clustering of data sets, e.g. to identify common chemotypes [2,3], and the identification of activity cliffs [4]. However, the choice of molecular description to calculate the similarity is not trivial and can vary depending on the compound selection and/or target [5-7]. A variety of descriptors exist which can be divided into two large groups depending if they consider only the 2D structure (topology) of a molecule or if they include 3D information. A standard and computationally efficient abstract representation is molecular fingerprints [8], where structural features are represented by either bits in a bit string or counts in a count vector. Fingerprints are compact and allow fast comparison of chemical structures. In this study, the focus is on 2D fingerprints and different algorithms to construct them are compared. The algorithms can be divided into four classes: (i) dictionary-based, (ii) topological or path-based fingerprints, (iii) circular fingerprints, and (iv) pharmacophores.

The performance of fingerprints is often tested in retrospective benchmarking studies using data sets made up of known actives and known or assumed inactives, so-called decoys. The performance of a fingerprint in these studies depends not only on its ability to describe the molecular features responsible for activity against a specific target, but also on the composition of the data set, the statistical robustness of the study, and the evaluation method(s) [9-14]. The data-set composition plays a central role when evaluating the differentiation of actives and inactives by a fingerprint [12]. If the actives in the data set belong to the same chemical series or have very different molecular properties, e.g. molecular weight or number of heavy atoms, than the inactives, it is easy for most fingerprints to distinguish actives from inactives. On the other hand, if the actives are very diverse, i.e. have different scaffolds, or if the actives and inactives have very similar molecular properties, fingerprints can have difficulties to differentiate actives from inactives. To help avoid being mislead by the first type of bias, it is advisable to use some sort of negative control, e.g. a simple atom count, to provide a baseline that the performance of more sophisticated fingerprints can be compared to [15].

In order to draw statistically robust conclusions from a benchmarking study of virtual screening, several issues need to be considered. For example, the number and diversity of protein targets, actives and inactives has to be large enough [10], and an error estimate of the performance should be obtained by boot-strapping, i.e. by repeating the ranking experiment many times with different random subsets of the actives as query molecules [11].

Just as there is wide variety of fingerprinting algorithms, there are multiple methods for evaluating VS performance and little consensus as to which is best [9,11]. The area under the receiver operating characteristic (ROC) curve (AUC) is widely used, as is the enrichment factor (EF) at a given fraction *χ* of the data set. The advantage of AUC is that it is bounded, running from 0 to 1 with 0.5 corresponding to randomness, and that it is independent of the ratio of actives to inactives and other external parameters. However, the AUC method has been critized as being inappropriate for comparing VS methods as it is not sufficiently sensitive to early recognition [9]. The EF explicitly measures early recognition but it is dependent on the ratio of actives to inactives and the choice of *χ*. To try and overcome these limitations numerous other evaluation methods, such as robust initial enhancement (RIE) [16] and Boltzmann-enhanced discrimination of ROC (BEDROC) [9], have been proposed. The RIE uses a continuously decreasing exponential weight as a function of rank and is thus sensitive to early recognition. It is, however, dependent both on an adjustable parameter, the exponential weight, and the ratio of actives to inactives. RIE values can therefore not be easily compared between different data sets. The BEDROC is constructed by, in essence, forcing the RIE to be bounded by 0 and 1, avoiding the dependence on the active/inactive ratio. In this contribution, all of these methods are used, the results are compared and correlations are investigated.

In order to be able to compare results from benchmarking studies, it is important to use standard data sets that are readily available to other researchers. One of the first collections used in multiple benchmarking studies was data for eleven targets taken from the MDL Drug Data Report (MDDR) [13,15,17-21] However, the MDDR, a commercial product, places limits on redistribution of the data, hindering the development of an open validation set. Two recently developed and publicly available compound data sets do not have this problem: the directory of useful decoys (DUD) [22,23] and the maximum unbiased validation (MUV) data sets [24,25]. ChEMBL [26,27] also provides a rich source of data: a recent publication presented a subset of targets from ChEMBL for use in VS experiments [28]. All three of these collections of open data sets have been used in recent benchmarking studies [3,28-33].

In these benchmarking studies, no globally best fingerprinting method has been found. However, some general trends have been observed. Using multiple actives as query molecules together with some kind of data fusion [34] has been found to enhance VS performance [18,29,35]. Different studies found that 2D methods generally outperform 3D methods [13,20,31-33]. Although *inter*-target differences are greater than differences between fingerprints for a single target, Bender *et al.*[15] could identify four large groups with similar performance based on a principal-component analysis: (i) path-based fingerprints or predefined keys, (ii) circular fingerprints using bit strings, (iii) circular fingerprints using count vectors, and (iv) pharmacophores, where circular fingerprints showed overall a good performance. This is in agreement with a study from Hert *et al.*[19] where circular fingerprints were found to be more effective than the other fingerprint types. In another study the effect of the length of the bit string and thus the effect of collisions was investigated, leading to the conclusion that longer bit strings perform better [21]. Note that each of these publications use different sets of fingerprints, different reference data sets, and different evaluation criteria, making it impossible to directly compare their results and conclusions.

The existence of publicly available data sets and detailed descriptions of benchmarking studies in the literature were a first step towards comparable results of VS methods. However, the exact results may depend on implementation details of the computational procedure and the random processes used, such as the selection of the query molecules. Here, we go a step further towards true reproducibility and comparability by providing data sets and source code which can be reused for future comparisons. The benchmarking platform described below contains lists of the actives and inactives used from three different data-set collections (DUD, MUV and ChEMBL), lists of randomly selected query molecules for VS runs, and python scripts used for the simulated virtual screening and scoring. In addition, the results for a set of 14 2D fingerprints belonging to three of the four descriptor classes are shown using multiple different evaluation methods. The fingerprints themselves were also generated using open-source software, so the results in this study should be fully reproducible by any researcher.

## Results and discussion

Using the benchmarking platform, the performance of 14 2D fingerprints covering dictionary-based, path-based and circular fingerprints was assessed over 88 targets from three publicly available collections of data sets. To ensure robust statistics, 50 repetitions, each with five query molecules, of the VS experiment were performed.

### Performance for 88 targets

**Figure 1:**
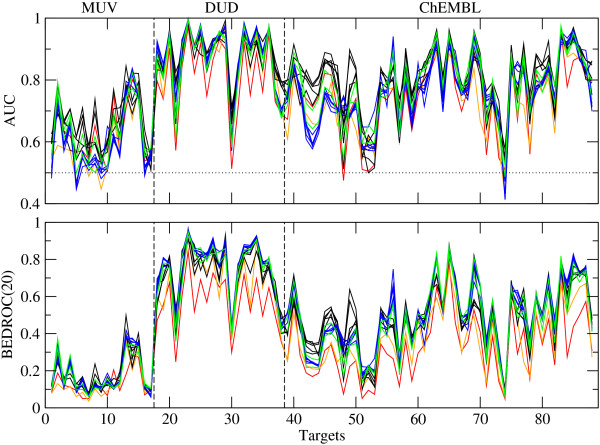
**Performance of 14 fingerprints.** Average performance of 14 2D fingerprints measured with AUC (top) and BEDROC(*α*=20) (bottom). The two baseline fingerprints are shown in red (ECFC0) and orange (MACCS). Path-based fingerprints (AP, TT, Avalon, long Avalon, RDK5) are shown in black, circular fingerprints with bit string (ECFP4, long ECFP4, ECFP6, long ECFP6, FCFP4) in blue, and circular fingerprints with count vector (ECFC4, FCFC4) in green. The horizontal, dotted line indicates random distribution.

The performance of 14 2D fingerprints measured with AUC and BEDROC(*α*=20) is shown in Figure 1. The two methods show the same trends over the targets, as do the other evaluation methods (data not shown). The data for all tested evaluation methods are provided as csv files in Additional file 1.

As can be seen from Figure 1, the performance of all fingerprints is poor for the MUV data sets, which has also been found by Tiikkainen *et al.*[29]. There are four targets, i.e. 548 (PKA), 832 (cathepsin G), 846 (Factor XIa) and 852 (Factor XIIa), which give some enrichment of actives. However, for other targets the performance is worse than random for some fingerprints. The baseline fingerprints, ECFC0 and MACCS, show no worse performance than the other, higher-level fingerprints for these data sets.

The performance of the fingerprints is generally very good for the DUD data sets, except for CDK2 and HIVRT, and to a lesser extent VEGFR2, but all show AUC values above random. The baseline fingerprint, ECFC0, shows a clearly worse performance than the other fingerprints. The targets DHFR, ER_agonist, GR and SAHH are rather too easy for this type of VS experiment: they have an AUC value above 0.95 for many of the fingerprints, including the baseline MACCS fingerprint.

Heikamp and Bajorath selected the ChEMBL targets with minimum 30% compound recovery rate (RR) for MACCS and maximum 80% compound RR for ECFP4 [28]. As they used all available actives and 1 million randomly selected decoys, whereas only the 100 most diverse actives and 10000 decoys with 0.5 similarity to at least one active are used in this study, the results here differ somewhat. The targets of the ChEMBL collection show different difficulty levels. For one target, 12911 (cytochrome P450 2C9), the performance of many fingerprints is below random, and for other targets, i.e. 12209 (carbonic anhydrase XII), 43 (*β*-2 adrenergic receptor), 219 (M3 receptor) and 130 (D3 receptor), it is close to random. On the other hand, the AUC value of some fingerprints is above 0.95 for the targets 11265 (Somatostin receptor 5), 12679 (C5a receptor), 237 (Leukotriene A4 hydrolase), 10927 (Urotensin II receptor), 11442 (liver glycogen phosphorylase). The baseline fingerprints have the lowest AUC values in most cases and the lowest BEDROC values for nearly all targets.

In general, the differences between fingerprints for a given target are smaller than the differences between targets for a given fingerprint. This is important to keep in mind as the performance of the fingerprints across targets is evaluated.

### Correlations between evaluation methods

**Figure 2:**
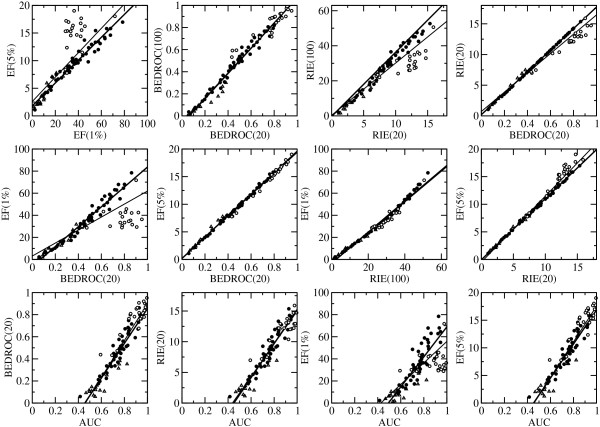
**Correlations between evaluation methods.** A selection of 12 correlations between evaluation methods using the average score of 2D fingerprint ECFP4 for the three collection of data sets: MUV (open triangles), DUD (open circles), and ChEMBL (filled circles). The maximum value is 100.0 for EF(1%), and 20.0 for EF(5%). For RIE, the maximum value of the ChEMBL data sets was used, i.e. 18.1 for RIE(20), and 63.2 for RIE(100). Linear regression curve is shown for all 88 targets (thin line) and for the 67 targets of MUV and ChEMBL (thick line).

A selection of 12 out of 21 possible correlations between the evaluation methods for the fingerprint ECFP4 is shown in Figure 2 together with the lines obtained from linear regression. The numerical values of the slope and constant, the correlation coefficient *r*, the coefficient of determination *r*^2^, and the root-mean-square error (RMSE) are given in Additional file 2: Table S1.

In general, the correlation between the methods is very high, as can be expected from their relationship [9]. The outliers which can be observed are for methods that are dependent on the actives/decoys ratio, i.e. RIE(20), RIE(100) and EF(1%). These appear in the DUD data sets, which have varying number of actives and decoys. EF(5%) shows no outliers as all data sets have an actives/decoys ratio smaller than 0.05. 1/ *α* for BEDROC and RIE has a similar meaning to *χ* for EF [9], which can be seen in the panels in the middle row of Figure 2. If BEDROC(20) is compared to EF(1%), outliers can be observed for the DUD data sets (*r*^2^(all) = 0.668 and *r*^2^(without DUD) = 0.964), whereas there is an almost perfect correlation to EF(5%) (*r*^2^(all) = 0.995 and *r*^2^(without DUD) = 0.996). The same is observed between RIE(20) and EF(5%). If EF(1%) is compared to RIE(100) (or BEDROC(100), not shown), on the other hand, there is also a very strong correlation and no outliers (*r*^2^(all) = 0.980 and *r*^2^(without DUD) = 0.988). Interestingly, the DUD data sets obtain medium RIE(100) and EF(1%) although they have very high AUC or EF(5%) values, because the maximum value depends on the actives/decoys ratio (and gets smaller as the actives/decoys ratio gets larger) for these data sets. This makes it difficult to directly compare the values for different targets, and it shows clearly that the external parameters of the evaluation methods have to be chosen carefully. The fraction *χ* for EF should be smaller than the ratio actives/decoys, and the condition $\alpha \frac{\text{actives}}{\text{decoys}}\;\ll \;1$ should be met for RIE [9].

AUC is the only evaluation method discussed here which reflects the performance over the whole data set. It is therefore not as closely related to the other methods as these are with each other. Nevertheless, there are clear correlations between AUC and the other evaluation methods if they are not or only weakly dependent on the actives/decoys ratio, i.e. EF(5%) (*r*^2^(all) = 0.908), BEDROC(20) (*r*^2^(all) = 0.894) and RIE(20) (*r*^2^(all) = 0.899). A random AUC-value of 0.5 corresponds to approximately zero in all the other methods.

The observations discussed above indicate that the differences between the VS performance-evaluation methods are small, especially between the “early recognition” methods. The least convenient method is probably RIE as the maximum value is always dependent on the actives/decoys ratio, even if *α* is chosen to be sufficiently small. It is recommended, therefore, to provide both values for AUC and one of the “early recognition” methods, EF or BEDROC, with appropriate parameters for future benchmarking studies.

### Correlations between fingerprints

**Figure 3:**
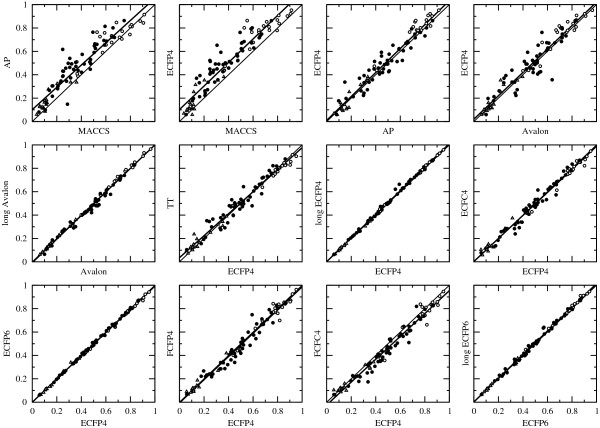
**Correlations between fingerprints.** A selection of 12 correlations between 2D fingerprints using the average score of evaluation method BEDROC(20) for the three collection of data sets: MUV (open triangles), DUD (open circles), and ChEMBL (filled circles). The thin line corresponds to *y* = *x* while the linear regression curve is shown as a thick, black line.

A selection of 24 out of 91 possible correlations between fingerprints for the evaluation method BEDROC(20) is shown in Figure 3 and Additional file 2: Figure S1 together with the linear regression curves. The numerical values of the slope and constant, the correlation coefficient *r*, the coefficient of determination *r*^2^, and the root-mean-square error (RMSE) are given in Additional file 2: Table S2.

Although the fingerprints belong to three different classes, all show medium to strong correlations between them. The largest scattering can be observed when the comparison involves ECFC0 and MACCS (*r*^2^ between 0.88 and 0.90), or path-based fingerprints such as AP (*r*^2^ between 0.91 and 0.97). The circular fingerprints show strong correlations between the different variants (*r*^2^ between 0.97 and 1.0), with ECFP4 and ECFP6 having the strongest (*r*^2^ = 0.999). Across this wide range of data sets, these two common choices for the circular-fingerprint radius are equivalent. In addition, the length of the bit string has a small effect as seen in the very strong correlation between the 1024-bits and 16384-bits version of ECFP4 (*r*^2^ = 0.998), ECFP6 (*r*^2^ = 0.996), and also Avalon (*r*^2^ = 0.993). For these three fingerprints, no considerable improvement of the performance with increased bit space could be observed, which deviates from an earlier finding of Sastry *et al.*[21] for Daylight-like fingerprints. Comparing the number of bits set in the short and long version of ECFP4 and ECFP6 (data not shown) revealed that the number of collisions introduced by folding from 1024 bits to 16384 bits was very low. In the case of the Avalon fingerprint, more collisions were observed, but overall the number was still low and similarities were not strongly affected. Thus, performance differences observed in the earlier study are likely due to the use of a different hashing function and the higher bit density of the Daylight-like linear fingerprints used leading to more collisions.

### Ranking of fingerprints

**Figure 4:**
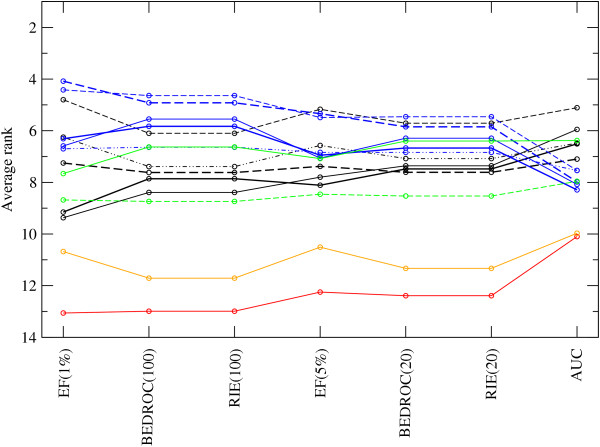
**Average rank across 88 targets of the 14 2D fingerprints.** Baseline fingerprints are shown in red (ECFC0) and orange (MACCS), path-based fingerprints in black, i.e. AP (fine, solid), TT (fine, dashed), Avalon (thick, solid), long Avalon (thick, dashed), RDK5 (fine, dashed-dotted), circular fingerprints with bit strings in blue, i.e. ECFP4 (fine, solid), long ECFP4 (fine, dashed), ECFP6 (thick, solid), long ECFP6 (thick, dashed), FCFP4 (fine, dashed-dotted), and circular fingerprints with counts in green, i.e. ECFC4 (solid), FCFC4 (dashed).

For each of the 50 repetition of the VS experiment, the 14 fingerprints were ranked according to their performance. These ranks can be averaged across repetitions and targets (Figure 4, and Additional file 2: Table S3). As the standard deviation of the average rank is still large, a global Friedman test was performed as a first step to assess if certain fingerprints are consistently better than another. The p-value of the global Friedman test was below 2.2 ·10^−16^ for each evaluation method indicating that there are statistically significant differences between fingerprints and that a post-hoc analysis is required.

In a second step, 91 post-hoc pairwise Friedman tests were performed to determine which pairs of fingerprints show a statistically significant difference. Note, however, that statistically significant differences may not always mean practically meaningful differences [36]. The results are shown as a matrix for AUC and BEDROC(20) in Table 1 where the fingerprints in each table are ordered by increasing average rank (low ranks are better). If all 100 resampled, adjusted p-values are below the confidence level *α* of 0.05, which indicates a statistically significant difference between the two fingerprints, an “-” is inserted in the matrix. If all 100 p-values are above *α*, an “X” is used. An “o” represents the distribution of adjusted p-values above and below *α*. The numerical values of the resampled, adjusted p-values are given for all evaluation methods as csv files in Additional file 3.

**Table 1:** Pairwise post-hoc Friedman tests

	**TT**	**AP**	**ECFC4**	**RDK5**	**Avalon**	**lAvalon**	**FCFP4**	**lECFP4**	**FCFC4**	**lECFP6**	**ECFP4**	**ECFP6**	**MACCS**	**ECFC0**	**Rank**
**TT**		X	X	X	X	-	-	-	-	-	-	-	-	-	1
**AP**			X	X	X	X	o	o	-	-	-	-	-	-	1
**ECFC4**				X	X	X	X	X	o	o	o	-	-	-	1
**RDK5**					X	X	X	X	X	o	o	-	-	-	1
**Avalon**						X	X	X	X	o	o	-	-	-	1
**lAvalon**							X	X	X	X	X	X	-	-	1
**FCFP4**								X	X	X	X	X	-	-	1
**lECFP4**									X	X	X	X	-	-	1
**FCFC4**										X	X	X	o	-	1
**lECFP6**											X	X	o	o	1
**ECFP4**												X	o	o	1
**ECFP6**													X	o	1
**MACCS**														X	1
**ECFC0**															1
	**lECFP4**	**TT**	**lECFP6**	**ECFP4**	**ECFC4**	**ECFP6**	**FCFP4**	**RDK5**	**AP**	**Avalon**	**lAvalon**	**FCFC4**	**MACCS**	**ECFC0**	**Rank**
**lECFP4**		X	X	X	X	o	o	o	-	-	-	-	-	-	1
**TT**			X	X	X	X	X	X	o	o	-	-	-	-	1
**lECFP6**				X	X	X	X	X	o	o	o	-	-	-	1
**ECFP4**					X	X	X	X	X	X	X	-	-	-	1
**ECFC4**						X	X	X	X	X	X	-	-	-	1
**ECFP6**							X	X	X	X	X	o	-	-	1
**FCFP4**								X	X	X	X	-	-	-	1
**RDK5**									X	X	X	o	-	-	1
**AP**										X	X	o	-	-	1
**Avalon**											X	X	-	-	1
**lAvalon**												X	-	-	1
**FCFC4**													-	-	1
**MACCS**														X	13
**ECFC0**															13

Results from pairwise post-hoc Friedman tests of the average rank between 14 2D fingerprints for the evaluation methods AUC (top) and BEDROC(20) (bottom). Pairs of fingerprints with no statistically significant difference are marked with “X”, pairs with an adjusted p-value distribution around the confidence level *α* with “o”, and pairs with a statistically significant difference with “-”. Fingerprints are ordered according to ascending average rank.

In AUC, there is no clear separation between groups or individual fingerprints as can be expected from Figure 4. This is true even of the baseline fingerprints. It is possible to conclude, however, that path-based fingerprints such as TT and AP (and to some extent also RDK5 and Avalon) are ranked higher than the circular fingerprints, with the exception of ECFC4. In BEDROC(20), the two baseline fingerprints, ECFC0 and MACCS, are ranked lower from the other fingerprints. Between the other fingerprints, there is again no significant separation, although circular fingerprints (especially long ECFP4 and long ECFP6) are - in contrast to AUC - ranked statistically significantly higher than path-based fingerprints. The other “early recognition” methods show a similar picture (Additional file 2: Tables S4 and S5). The only exception to this trend is TT: TT is ranked among the top fingerprints in all evaluation methods. This is remarkable as the topological torsion fingerprint is rather simple and is one of the oldest, published in 1987, descriptors considered here [37].

### Analysis of scaffold diversity

**Figure 5:**
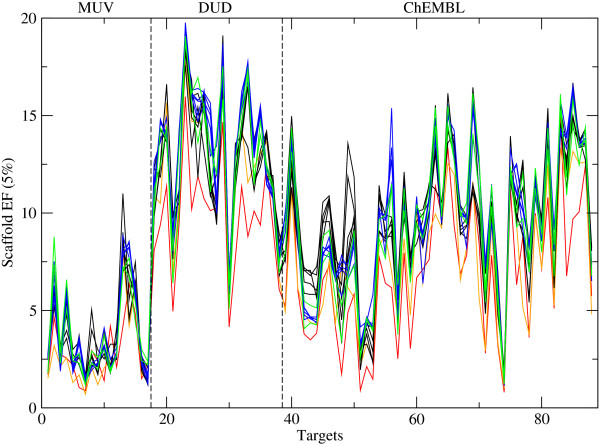
**Scaffold enrichment factor of 14 fingerprints.** Average scaffold enrichment factor (scaffold EF) at 5% for 88 targets. The two baseline fingerprints are shown in red (ECFC0) and orange (MACCS). Path-based fingerprints (AP, TT, Avalon, long Avalon, RDK5) are shown in black, circular fingerprints with bit string (ECFP4, long ECFP4, ECFP6, long ECFP6, FCFP4) in blue, and circular fingerprints with count vector (ECFC4, FCFC4) in green.

**Figure 6:**
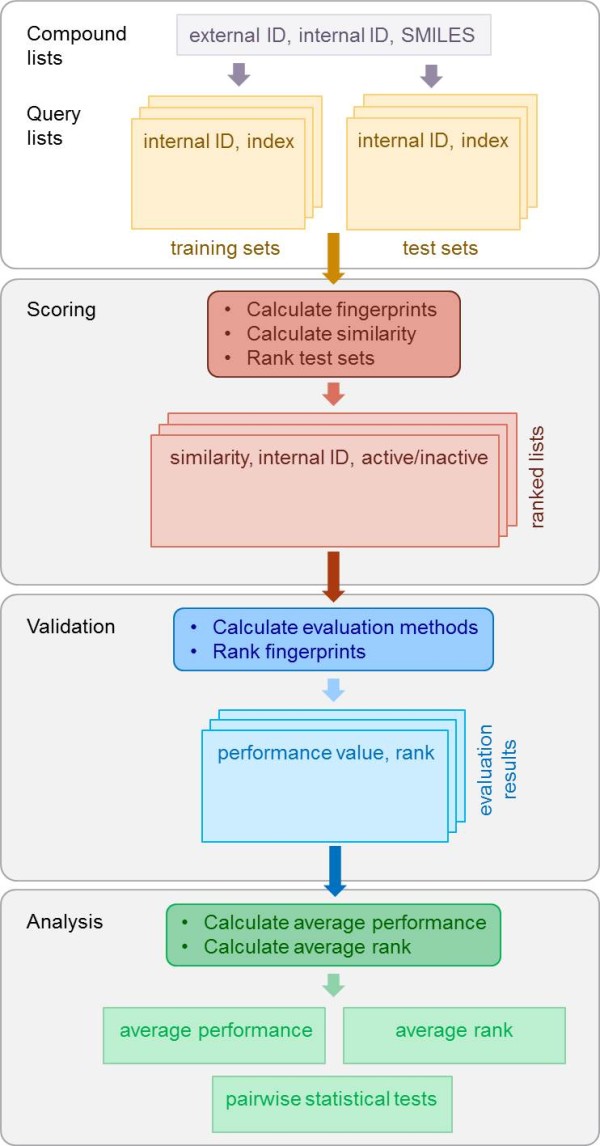
**Scaffold enrichment factor versus enrichment factor.** Average ratio of scaffold enrichment factor (scaffold EF) and the enrichment factor (EF) at 5% of six fingerprints, ECFC0, AP, TT, ECFP4, FCFC4 and lECFP6, as a function of the ratio BMS/actives for the three collection of data sets: MUV (open circles), DUD (open triangles), and ChEMBL (filled circles). The black, dashed line corresponds to *y* = 1.0.

In VS, the ability to recognize structurally diverse but functionally similar molecules, called “scaffold hopping”, is considered to be a desirable property of a similarity method [38]. Although 2D fingerprints are simple similarity methods, some have been found to have a significant scaffold-hopping potential [39,40]. Unfortunately, the definition of a scaffold is ambiguous and there exists a range of definitions [38], where the Bemis-Murcko scaffolds (BMS) [41] of molecular frameworks is widely adopted. Because the BMS definition has been used in studies assessing the scaffold-hopping potential of 2D fingerprints [39,40] it is used here. The number of BMS found among the actives of the 88 data sets and the ratio BMS/actives are given in Table 2. The average performance of ECFP4 determined by AUC and BEDROC(20) as a function of the ratio BMS/actives is shown in Additional file 2: Figure S2. A negative exponential relationship can be observed between the performance and the ratio BMS/actives in the data set. Data sets with few distinct scaffolds among the actives, i.e. BMS/actives < 50%, tend to be fairly easy for similarity-based VS, so nearly all actives are ranked at the beginning of the list and nearly all (of the few) scaffolds are found, making the assessment of the scaffold-hopping potential pointless. On the other hand, if there are many distinct scaffolds, i.e. BMS/actives = 80 - 100%, nearly every active found will correspond to a new scaffold, which also makes the assessment of the scaffold-hopping potential pointless. The scaffold-hopping potential can be measured using the scaffold EF, which is calculated analogously to EF, i.e. the number of scaffolds retrieved among the first 5%, i.e. *χ* = 0.05, divided by the number of scaffolds in a random distribution, i.e. *χ*_*BMS*_[3]. The scaffold EF as a function of the targets is shown in Figure 5. Again the differences among the fingerprints are smaller than the differences between targets for a single fingerprint, and Figure 5 is generally very similar to Figure 1. This implies that, at least for the range of data sets considered here, the scaffold EF is closely related to the general performance of the fingerprint, not only for the extreme cases of BMS/actives > 80% and < 50% but in general. This is indeed true for the majority of targets as can be seen in Figure 6 where the ratio scaffold EF/EF at 5% is close to 1.0 for most targets independent of the ratio BMS/actives.

**Table 2:** Information of data sets

**Origin**	**Target ID**	**Description**	**A**	**D**	**A/D**	**BMS**	**B/A**
MUV	466	Sphingosine 1-phosphate (S1P1) receptor	30	15000	0.002	30	1.00
	548	Protein kinase A (PKA)	30	15000	0.002	29	0.97
	600	Steroidogenic factor 1 (SF1): inhibitors	30	15000	0.002	29	0.97
	644	Rho-kinase 2	30	15000	0.002	28	0.93
	652	HIV-1 RT-Rnase H	30	15000	0.002	29	0.97
	689	Ephrin receptor A4	30	15000	0.002	30	1.00
	692	Steroidogenic factor 1 (SF1): agonists	30	15000	0.002	30	1.00
	712	Heat shock protein 90 (HSP90)	30	15000	0.002	27	0.90
	713	Estregon receptor (ER) *α*: inhibitors	30	15000	0.002	29	0.97
	733	Estregon receptor (ER) *β*	30	15000	0.002	30	1.00
	737	Estregon receptor (ER) *α*: potentiators	30	15000	0.002	29	0.97
	810	Focal adhesion kinase (FAK)	30	15000	0.002	28	0.93
	832	Cathepsin G	30	15000	0.002	25	0.83
	846	Factor XIa (FXIa)	30	15000	0.002	24	0.80
	852	Factor XIIa (FXIIa)	30	15000	0.002	24	0.80
	858	Dopamin receptor D1	30	15000	0.002	27	0.90
	859	Muscarinic receptor M1	30	15000	0.002	30	1.00
DUD	ace	Angiotensin-converting enzyme	46	1796	0.026	28	0.61
	ache	Acetylcholin esterase	99	3859	0.026	37	0.37
	ar	Androgen receptor	68	2848	0.024	21	0.31
	cdk2	Cyclin-dependent kinase 2	47	2070	0.023	39	0.83
	cox2	Cyclooxygenase-2	212	12606	0.011	97	0.46
	dhfr	Dihydrofolate reductase	190	8350	0.023	42	0.22
	egfr	Epidermal growth factor receptor	365	15560	0.023	86	0.24
	er_agonist	Estregon receptor (ER): agonists	63	2568	0.025	26	0.41
	fgfr1	Fibroblast growth factor receptor	71	3462	0.021	18	0.25
	fxa	Factor Xa	64	2092	0.031	28	0.44
	gpb	Glycogen phosphorylase	49^1^	21321	0.023	14	0.29
	gr	Glucocorticoid receptor	32	2585	0.010	14	0.44
	hivrt	HIV-1 RT-Rnase	34	1494	0.023	25	0.74
	inha	Enoyl reductase	57	2707	0.021	35	0.61
	na	Neuramidase	49	1713	0.029	13	0.27
	p38	P38 MAP kinase	137	6779	0.020	47	0.34
	parp	Poly(ADP-ribose) polymerase	31	1350	0.023	9	0.29
	pdgfrb	Platelet-derived growth factor receptor *β*	124	5603	0.022	38	0.31
	sahh	*S*-adenosylhomocysteine hydrolase	33	1344^2^	0.025	10	0.30
	src	Tyrosine-protein kinase C-SRC	98	5679	0.011	30	0.31
	vegfr2	Vascular endothelial growth factor receptor 2	48	2712	0.011	39	0.81
ChEMBL	11359	Phosphodiesterase 4D	100	10000	0.010	81	0.81
	28	Thymidylate synthase	100	10000	0.010	48	0.48
	11536	Ghrelin receptor	100	10000	0.010	87	0.87
	8	Tyrosine-protein kinase ABL	100	10000	0.010	93	0.93
	10434	Tyrosine-protein kinase SRC	100	10000	0.010	94	0.94
	12670	Tyrosine-protein kinase receptor FLT3	100	10000	0.010	95	0.95
	20014	Serine/threonine-protein kinase Aurora-A	100	10000	0.010	87	0.87
	234	Insulin-like growth factor I receptor	100	10000	0.010	86	0.86
	12261	c-Jun N-terminal kinase I	100	10000	0.010	65	0.65
	12209	Carbonic anhydrase XII	100	10000	0.010	66	0.66
	25	Glucocorticoid receptor	100	10000	0.010	87	0.87
	36	Progesterone receptor	100	10000	0.010	78	0.78
	43	*β*-2 adrenergic receptor	100	10000	0.010	94	0.94
	219	Muscarinic acetylcholine receptor M3	100	10000	0.010	94	0.94
	130	Dopamine receptor D3	100	10000	0.010	92	0.92
	105	Serotonin 1d (5-HT1d) receptor	100	10000	0.010	66	0.66
	11336	Neuropeptide Y receptor type 5	100	10000	0.010	77	0.77
	20174	G protein-coupled receptor 44	100	10000	0.010	77	0.77
	126	Cyclooxygenase-2	100	10000	0.010	88	0.88
	11225	Renin	100	10000	0.010	81	0.81
	12252	*β*-secretase 1	100	10000	0.010	93	0.93
	11682	Glycine transporter 1	100	10000	0.010	76	0.76
	134	Vasopressin V1a receptor	100	10000	0.010	87	0.87
	116	Oxytocin receptor	100	10000	0.010	64	0.64
	11265	Somatostatin receptor 5	100	10000	0.010	59	0.59
	10475	Neuropeptide Y receptor type 1	100	10000	0.010	45	0.45
	12679	C5a anaphylatoxin chemotactic receptor	100	10000	0.010	45	0.45
	10579	C-C chemokine receptor type 4	100	10000	0.010	64	0.64
	11575	C-C chemokine receptor type 2	100	10000	0.010	80	0.80
	18061	Sodium channel protein type IX *α* subunit	100	10000	0.010	62	0.62
	237	Leukotriene A4 hydrolase	100	10000	0.010	65	0.65
	276	Phosphodiesterase 4A	100	10000	0.010	70	0.70
	11534	Cathepsin S	100	10000	0.010	91	0.91
	10198	Voltage-gated potassium channel subunit Kv1.5	100	10000	0.010	69	0.69
	10498	Cathepsin L	100	10000	0.010	90	0.90
	12911	Cytochrome P450 2C9	100	10000	0.010	96	0.96
	12968	Orexin receptor 2	100	10000	0.010	36	0.36
	100579	Nicotinic acid receptor 1	100	10000	0.010	70	0.70
	100126	Serine/threonine-protein kinase B-raf	100	10000	0.010	78	0.78
	10378	Cathepsin B	100	10000	0.010	83	0.83
	10417	P2X purinoceptor 7	100	10000	0.010	65	0.65
	10752	Inhibitor of nuclear factor *κ* B kinase *β* subunit	100	10000	0.010	64	0.64
	10773	Interleukin-8 receptor B	100	10000	0.010	55	0.55
	11631	Sphingosine 1-phosphate receptor Edg-1	100	10000	0.010	77	0.77
	10927	Urotensin II receptor	100	10000	0.010	59	0.59
	11085	Melatonin receptor 1B	100	10000	0.010	71	0.71
	11442	Liver glycogen phosphorylase	100	10000	0.010	55	0.55
	11279	Metabotropic glutamate receptor 1	100	10000	0.010	65	0.65
	11488	Estradiol 17- *β*-dehydrogenase 3	100	10000	0.010	47	0.47
	12840	Macrophage colony stimulating factor receptor	100	10000	0.010	81	0.81

## Conclusions

An open-source platform to benchmark 2D fingerprints for virtual screening (VS) was developed and used to assess the performance of 12 commonly used fingerprints together with two baseline fingerprints.

The platform currently incorporates 88 targets from three publicly available collections of data sets: MUV, DUD and ChEMBL. The VS experiment is divided into three steps: scoring, validation and analysis. The platform uses the open-source cheminformatics toolkit RDKit to calculate fingerprints and similarities, but through the three-stage design data generated by other sources can easily be fed in at the validation or analysis stages. The platform with its compound and training lists and source code allows easy reproduction and comparison of the performance of 2D fingerprints.

The performance of 14 2D fingerprints (including two baseline fingerprints) and two different bit-string sizes for three fingerprints was assessed using five query molecules over 88 targets and four different evaluation methods with different parameters. Except for the baseline fingerprints, the performance of all fingerprints is generally similar. This finding is supported by the strong correlations observed between the fingerprints, especially between circular fingerprints with different diameter size and between fingerprints with different bit-string size. The *inter*-target difference in performance is greater than the *intra*-target difference between fingerprints, as has been also found by other studies. The MUV data sets were the most difficult of those studied here. In order to obtain a measure of the overall performance, the fingerprints were ranked for each VS experiment and the ranks were then averaged over the repetitions and targets. The baseline fingerprints, ECFC0 and MACCS, were indeed found to be statistically significantly ranked last of all fingerprints in this study using “early recognition” evaluation methods. Path-based fingerprints were generally ranked higher using the AUC method, whereas circular fingerprints are generally ranked higher by “early recognition” methods such as EF, BEDROC and RIE. The exception is the topological torsions fingerprint which is ranked among the top fingerprints by all evaluation methods.

There has been discussion in the literature about the correct evaluation method for simulated VS experiments. However, strong correlations were found between the different “early recognition” methods if appropriate parameters were used, i.e. the fraction *χ* for EF and the exponential weight *α* for BEDROC and RIE, respectively. Thus, we recommend to provide results from AUC and one of the “early recognition” methods for future benchmarking studies. The fraction parameter for EF is more immediately understandable than the exponential weight for BEDROC, but BEDROC has the advantage of running from 0 to 1.

Scaffold-hopping potential is considered an important ability for VS methods. Although 2D fingerprints are simple and based on similarity, they were found to have a significant potential to retrieve structurally diverse molecules. However, a strong correlation was observed between VS performance and scaffold enrichment factor (scaffold EF), which makes the assessment of the scaffold-hopping potential rather futile.

## Methods

### Cheminformatics toolkit

The benchmarking platform presented in this study uses the RDKit [42], an open-source cheminformatics toolkit made available under the permissive Berkeley Software Distribution (BSD) license.

### Fingerprints

Four classes of 2D fingerprint types can be distinguished: (i) dictionary-based, (ii) topological or path-based, (iii) circular fingerprints and (iv) pharmacophores. In addition, fingerprints can differ in the atom types or feature classes used or the length of the bit string. In this study, 14 fingerprints belonging to three of the four classes were compared.

The public Molecular ACCess System (MACCS) structural keys [43] are 166 predefined substructures defined as SMARTS and belong to the dictionary-based fingerprint class. They were originally designed for substructure search and typically show a low performance level for virtual screening, thus they are often used as baseline fingerprint for benchmarking studies.

Topological or path-based fingerprints describe combinations of atom types and paths between atom types. In atom pair (AP) fingerprints [44], pairs of atoms together with the number of bonds separating them are encoded. In topological torsions (TT) [37], on the other hand, four atoms forming a torsion are described. In both AP and TT fingerprints the atom type consists of the element, the number of heavy-atom neighbours and the number of *π*-electrons.

The RDKit fingerprint, a relative of the well-known Daylight fingerprint [45], is another topological descriptor. Atom-types, the atomic number and aromaticity state, are combined with bond types to hash all branched and linear molecular subgraphs up to a particular size [42]. In this study, a maximum path length of five (RDK5) was used.

Similar to the Daylight fingerprints, certain paths and feature classes of the molecular graph are enumerated and hashed in the Avalon fingerprint [46]. There are 16 feature classes which were optimized for substructure search. A detailed description of the feature classes is given in Table 1 and the supplementary material of [46].

Circular fingerprints were developed more recently [47], and encode circular atom environments up to a certain bond radius from the central atom. If atom types consisting of the element, the number of heavy-atom neighbours, the number of hydrogens, the isotope and ring information are used these fingerprints are called extended-connectivity (EC) fingerprints. Alternatively, pharmacophoric features can be used, yielding functional connectivity (FC) fingerprints. We consider two representations of the fingerprints, bit strings (FP) and count vectors (FC). This gives four types of circular fingerprints: extended-connectivity bit string (ECFP), extended-connectivity count vector (ECFC), feature-connectivity bit string (FCFP) and feature-connectivity count vector (FCFC). The maximum bond length or diameter is added at the end to the name. In this study, the four types of circular fingerprints with a diameter 4, i.e. ECFP4, ECFC4, FCFP4 and FCFC4, as well as ECFP6 were compared. In addition, ECFC0, which is a kind of atom count, was used as a second baseline fingerprint.

For all bit-string fingerprints, a size of 1024 bits was used. However, Sastry *et al.* found that such a small bit space may result in many collisions which can affect VS performance [21]. To investigate this effect a larger bit space, 16384 bits, was used for three fingerprints: long ECFP4 (lECFP4), long ECFP6 (lECFP6) and long Avalon (lAvalon).

All fingerprints were calculated using the RDKit.

### Evaluation methods

Throughout this section, *n* represents the number of actives in the test data set and *N* the total number of molecules in the test data set.

#### Receiver Operating Characteristic (ROC) curve

The ROC method originates from signal detection analysis and has been widely used across many disciplines. It is defined as the true positive rate (TPR) as a function of the false positive rate (FPR). The TPR is the number of actives at a given rank position divided by the number of actives, and the FPR is the number of inactives at a given rank position divided by the number of inactives.

From the ROC curve, the area under the curve (AUC) can be calculated. The discrete formula for a set of ranked molecules is given as follows

(1) $$\text{AUC}=\frac{1}{\text{nN}}\overset{N}{\underset{i=2}{\sum }}A_{i}(I_{i}-I_{i-1}),$$

where *A* the cumulative count of actives at rank position *i*, and *I* the cumulative count of inactives at rank position *i*. The AUC is non-parametric and is bounded by 0 and 1. An AUC value of 0.5 corresponds to random performance.

#### Enrichment Factor (EF)

The concept of the EF is straightforward, which made it a very popular evaluation method in VS. The EF at a fraction *χ* of the ranked test set is calculated as the number of actives found divided by the expected number of actives from a random ranking,

(2) $$\text{EF}(\chi )=\frac{\overset{n}{\underset{i=1}{\sum }}\delta (r_{i})}{\mathrm{\chi n}},\;\mathrm{with}\;\delta (r_{i})=\left\{\begin{array}{l} 1,\;r_{i}\leq \mathrm{\chi N}\\ 0,\;r_{i}>\mathrm{\chi N} \end{array}\right),$$

where *r*_*i*_ indicates the rank of the *i*th active. The minimum value of *EF*(*χ*) is 0 and the maximum value is 1/ *χ* if *χ*≥*n*/*N* and *N*/*n* otherwise. In this study, we calculated the EF at 1% (*χ* = 0.01) and 5% (*χ* = 0.05). For all data sets considered here (see below) *χ* = 0.05 is larger than *N*/*n* and thus the maximum value of EF(5%) is 20. For *χ* = 0.01, *N*/*n* is only smaller for the MUV and ChEMBL data sets, leading to a maximum value of EF(1%) of 100. For the DUD data sets the maximum value of EF(1%) will depend on the exact ratio of actives and inactives in the individual data sets.

#### Robust Initial Enhancement (RIE)

The RIE method was developed by Sheridan *et al.*[16] to circumvent a problem encountered with the enrichment factor: having large variations when a small number of actives are used. Similar to EF, RIE can be viewed as the sum of a “score” for each active divided by the expected sum of scores for a random distribution. Whereas the score is 1 in the case of EF, RIE uses a continuously decreasing exponential weight *α* as a function of rank

(3) $$\text{RIE}(\alpha )=\frac{\overset{n}{\underset{i=1}{\sum }}e^{-\alpha r_{i}/N}}{{\langle \overset{n}{\underset{i=1}{\sum }}e^{-\alpha r_{i}/N}\rangle }_{\text{random}}}.$$

Truchon and Bayly [9] have analytically calculated the random average and obtained an exact formula,

(4) $$\text{RIE}(\alpha )=\frac{N}{n}\frac{\overset{n}{\underset{i=1}{\sum }}e^{-\alpha r_{i}/N}}{\frac{1-e^{-\alpha }}{e^{\alpha /N}-1}}.$$

The minimum and maximum value of RIE are dependent on *n*, *N* and *α*, and given as follows [9],

(5) $$\begin{array}{ll} \text{RI}E_{\text{min}}(\alpha ) & =\frac{N}{n}\frac{1-e^{\mathrm{\alpha n}/N}}{1-e^{\alpha }}\; \end{array}$$

(6) $$\begin{array}{ll} \text{RI}E_{\text{max}}(\alpha ) & =\frac{N}{n}\frac{1-e^{-\mathrm{\alpha n}/N}}{1-e^{-\alpha }}\; \end{array}$$

Since the meaning of 1/ *α* in RIE is very close to the meaning of *χ* in EF, we used *α* = 20 and 100 in this study to compare the performance.

#### Boltzmann-Enhanced Discrimination of ROC (BEDROC)

The main disadvantage of the RIE method is its minimum and maximum value which are not intuitive and which vary from data set to data set. The BEDROC method, which forces RIE to be bounded by 0 and 1, has been developed to overcome this [9],

(7) $$\text{BEDROC}(\alpha )=\frac{\text{RIE}(\alpha )-\text{RI}E_{\text{min}}(\alpha )}{\text{RI}E_{\text{max}}(\alpha )-\text{RI}E_{\text{min}}(\alpha )}.$$

Again, we used *α* = 20 and 100 in this study to compare the performance with the EF measures.

### Compound data sets

Data sets from three different public sources were used. The directory of useful decoys (DUD) was originally designed for benchmarking of docking methods [22]. A subset was later extracted for the use in ligand-based virtual screening (VS) experiments [23,30]. The DUD contains 40 targets with 4–365 actives and 9–15560 decoys. The decoys were selected from the ZINC database [48] based on physical properties of the actives. In this study the 21 targets with more than 30 actives were used.

In a recent study, Heikamp and Bajorath [28] proposed a set of 50 human targets extracted from ChEMBL [26,27] for use in VS experiments. They selected actives which had at least 10 *μ*M potency for direct interactions (relationship type = D) with a confidence level = 9. The target classes contain 50–625 actives. One million randomly selected molecules from ZINC were used as decoys. This selection process was repeated with ChEMBL version 14 (Heikamp and Bajorath extracted the actives from ChEMBL version 9). Additionally, molecules with a molecular weight > 700 g mol ^−^1 and molecules containing metal ions were excluded. From each of these sets of actives, the 100 most diverse compounds were chosen using the diversity picker of RDKit [42] resulting in total 5000 actives. For each active, two decoys with a Dice similarity > 0.5 using a simple atom-count fingerprint (ECFC0) were randomly selected from ZINC, resulting in total 10000 decoys.

The maximum unbiased validation (MUV) data sets [24,25] are based on PubChem [49] bioactivity data. MUV consists of assay data from 17 targets, each with 30 actives and 15000 decoys. Actives were selected from confirmatory screens and were chosen to be maximally spread based on simple descriptors and embedded in decoys. The decoys were selected from a primary screen for the same target. In short, these data sets were designed to be difficult for VS methods.

An overview of the 88 data sets with the target IDs, target description, number of actives, number of decoys, ratio actives/decoys, number of Bemis-Murcko scaffolds (BMS) [41] found in the actives and ratio BMS/actives is given in Table 2.

### Benchmarking platform

The scripts of the benchmarking platform are written in Python and use the Python library of the RDKit [42]. However, the scripts are designed in such a way that ranked compound lists generated by other sources can be used at a later stage of the VS experiment. The python scripts of the platform are given in Additional file 4.

#### Preparation of compound and training lists

For each target of the three data-set collections MUV, DUD and ChEMBL, two compound lists are provided, one for the actives and one for the decoys. In the case of ChEMBL, only one decoy compound list is given as the same decoys were used for all targets. The compound list contains the external ID (from the MUV, DUD, ChEMBL, and ZINC collections), internal IDs and SMILES. Internal IDs are defined as follows: *[name of data set]_[target ID]_[A/D]_[number of compound]*, where *A* indicates actives and *D* decoys. The compound number is determined by the order the compounds are listed in the original files.

The VS experiment is repeated 50 times for each target, randomly selecting each time a set of actives and a set of the decoys as *training molecules*. The remaining actives and decoys form the *test molecules* for the VS experiment. The platform provides for each target and number of query molecules a collection of 50 training lists with the internal ID and list index of *training molecules*.

The compound lists and the training lists with five query molecules and 20% of the decoys are given in Additional files 5 and 6.

#### Simulated virtual screening

**Figure 7:**
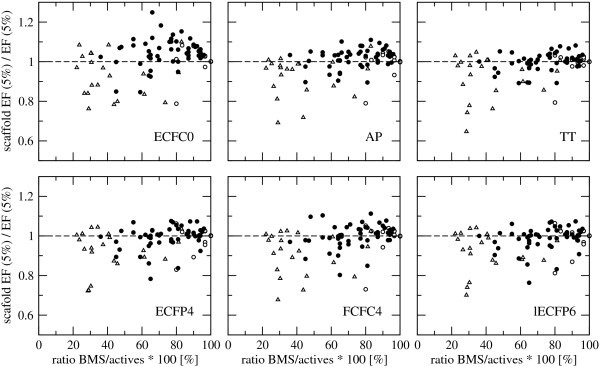
**Virtual screening scheme.** Schematic representation of the simulated virtual-screening (VS) experiment with the three steps scoring, validation and analysis.

The VS experiment is divided into the following three steps: scoring, validation and analysis. A schematic representation is given in Figure 7 and described below. The input and output of each step is given in Table 3. 

1) *Scoring*

The training lists are loaded. Fingerprints are calculated for all molecules, and the test molecules are ranked based on the similarity to the actives in the training set (the query molecules). Only the highest similarity value is considered for each test molecule, corresponding to the MAX fusion procedure [34]. Possible fingerprints are provided in an additional python script serving as the “fingerprint library”. Other fingerprints implemented in, or callable from Python can be added to this library. The default similarity measure used in this study is the Dice similarity. Other similarity measures which are currently supported by the platform are Tanimoto, Cosine, Russel, Kulczynski, McConnaughey, Manhattan and Rogot-Goldberg. The last two measures also take the common off-bits into account. The similarity measures are described and compared in [50]. The Dice and Tanimoto were found to give very similar results [50], which is expected given that the ranking of molecules provided by the two methods is provable to be exactly the same (see Appendix).

2) *Validation*

The ranked lists from the previous step are loaded and the performance of each fingerprint is calculated using different evaluation methods. The following evaluation methods are currently supported: AUC, RIE, BEDROC and EF.

3) *Analysis*

From the 50 ranked lists for each fingerprint and evaluation method, the average performance is calculated per target. In addition, the average rank of each fingerprint over all of the datasets is calculated per method. For each evaluation method, a global Friedman test [51] is performed to detect statistically significant differences between the mean ranks of the fingerprints.

**Table 3:** Arguments of virtual-screening scripts

**Step**	**Input (required)**		**Input (optional)**		**Output**
	**Key**	**Description**	**Key**	**Description**	**Description**
Scoring	-f	file containing fp names	-o	output path	for each target, a list
			-a	append the output file	with (fp name,
					list of 50 scored lists);
	-n	number of query molecules	-s	similarity measure (default: Dice)	each scored list
					contains (similarity,
					internal ID, active/inactive)
Validation	-m	file containing the evaluation	-o	output path	for each target,
		methods and their parameters	-i	input path(s)	a dictionary of methods;
			-r	file containing fingerprints to ignore	for each method,
					a dictionary of fps;
					for each fp, a list with
					(performance value, rank)
Analysis			-o	output path	for each target, a table
			-i	input path	with average performance per fp
					(columns) and method (rows);
					a file with average rank per fp
					(columns) and method (rows);
					a file with pairwise tests

Input and output arguments for the three steps of the virtual-screening (VS) experiment. Fingerprint is abbreviated by fp.

The Friedman test is a non-parametric alternative to an analysis of variance (ANOVA) for repeated measures by ranks. For each measure (i.e. target), the fingerprints are ranked based on the average rank over the 50 repetitions, and then for each fingerprint the ranks are summed. These sums are used to generate a p-value [51]. A p-value smaller than the confidence level *α* indicates that at least one method is consistently ranked higher than the others. Here, a confidence level *α* of 0.05 is used. If the p-value is significant, post-hoc pairwise Friedman tests [52] are performed to determine which method(s) is ranked consistently higher than the others. The post-hoc tests are performed using bootstrapping where the mean rank of each fingerprint per target and the average over all ranks are recalculated 100 times from a resampled set (with replacement) of the original 50 ranks. The p-values are multiplicity-adjusted using a non-parametric resampling method [53] (max T, part of the R package *multtest*) which allows the straightforward comparison of the resulting p-values with the confidence level *α*. Finally, the distribution and range of the bootstrapped p-values of the pairwise tests are used to categorize the differences in performance of the corresponding fingerprints into three classes: highly significant, significant around *α*, not significant. The calculations for significance testing were done using the R software environment for statistical computing [54].

## Appendix

Proof that molecule *A* is more similar to molecule *B* than molecule *C* for both the Tanimoto and Dice similarity. The Tanimoto similarity is defined as follows,

(8) $$\text{Tanimoto}=\frac{N_{A\text{\&}B}}{N_{A}+N_{B}-N_{A\text{\&}B}},$$

where *N*_*i*_ is the number of on-bits in the fingerprint of molecule *i*, and *N*_*i*&*j*_ is the number of common on-bits in the fingerprints of molecules *i* and *j*. The Dice similarity is given as,

(9) $$\text{Dice}=\frac{2N_{A\text{\&}B}}{N_{A}+N_{B}},$$

with the same notation.

So, if the Tanimoto similarity between molecules *A* and *B* is higher than the one between molecules *A* and *C*, then

(10) $$\frac{N_{A\text{\&}B}}{N_{A}+N_{B}-N_{A\text{\&}B}}>\frac{N_{A\text{\&}C}}{N_{A}+N_{C}-N_{A\text{\&}C}},$$

which can be reformulated as,

(11) $$N_{A\text{\&}B}(N_{A}+N_{C}-N_{A\text{\&}C})>N_{A\text{\&}C}(N_{A}+N_{B}-N_{A\text{\&}B}),$$

and finally leads to,

(12) $$N_{A\text{\&}B}(N_{A}+N_{C})>N_{A\text{\&}C}(N_{A}+N_{B}).$$

For the Dice similarity, it is

(13) $$\frac{2N_{A\text{\&}B}}{N_{A}+N_{B}}>\frac{2N_{A\text{\&}C}}{N_{A}+N_{C}},$$

which can be reformulated as,

(14) $$N_{A\text{\&}B}(N_{A}+N_{C})>N_{A\text{\&}C}(N_{A}+N_{B}),$$

which corresponds to Equation (12).

## Competing interests

Both authors declare that they have no competing interests.

## Authors’ contributions

SR participated in the conception and design of the benchmarking platform, collected the data sets, developed the benchmarking platform, performed the virtual screening experiments, and drafted the manuscript. GL participated in the conception and design of the benchmarking platform and in the discussion of the results, and helped to draft the manuscript. Both authors read and approved the final manuscript.

## Supplementary Material

Additional file 1 **Numerical Values of Results.** The file results.zip contains the csv files with the numerical values of the results of the benchmarking study. Click here for file

Additional file 2 **Supplementary Figures and Tables.** The file supplementary.pdf contains the additional figures and tables mentioned in the text. Click here for file

Additional file 3 **Resampled p-Values.** The file pvalues.zip contains the resampled p-values from the statistical analysis. Click here for file

Additional file 4 **Source Code.** The file python_scripts.zip contains the source code of the benchmarking platform. Click here for file

Additional file 5 **Compound Data Sets.** The file compounds.tar.gz contains the compound list of each target with external ID (MUV, DUD or ChEMBL ID), internal ID (used in benchmarking platform) and SMILES. Click here for file

Additional file 6 **Training Lists.** The file training_lists_5.tar.bz2 contains the indices of the compounds used for training for each target using five query molecules. Click here for file
