# Supplementary material for: Open-source platform to benchmark fingerprints for ligand-based virtual screening
Source: J Cheminform. 2013 May 30;5:26. doi: 10.1186/1758-2946-5-26 (PMC3686626; doi:10.1186/1758-2946-5-26)
Supplement: Additional file 2 — Supplementary Figures and Tables. The file supplementary.pdf contains the additional figures and tables mentioned in the text. [file 1758-2946-5-26-S2.pdf]

Open-Source Platform to Benchmark Fingerprints for Ligand-Based Virtual Screening

Sereina Riniker[1] and Gregory A. Landrum*[1]

[1] Novartis Institutes for BioMedical Research, Basel, Switzerland

Email: Sereina Riniker - sereina.riniker@novartis.com; Gregory A. Landrum* - gregory.landrum@novartis.com;

*Corresponding author

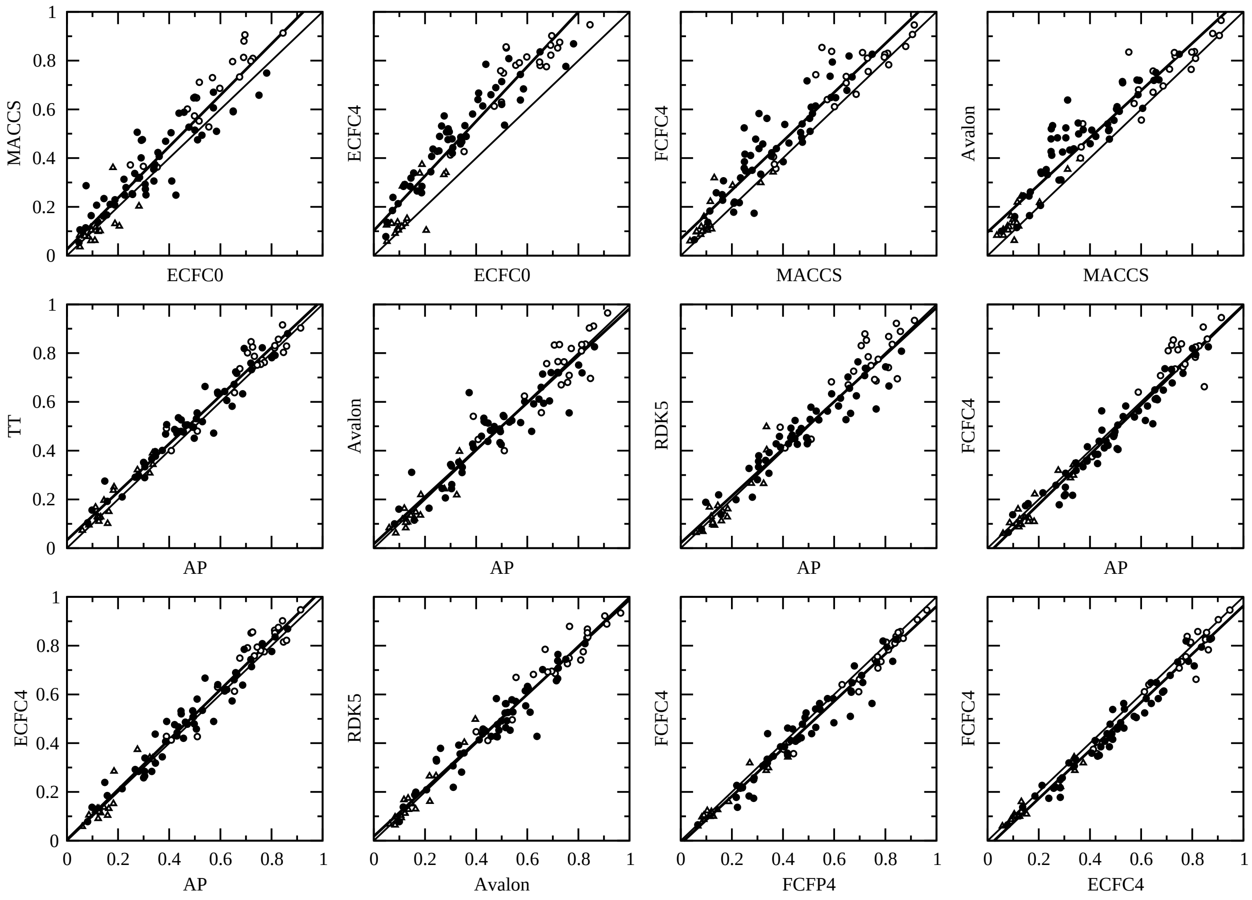

Figure S1: A selection of 12 correlations between 2D fingerprints using the average score of evaluation method BEDROC(20) for the three collection of data sets: MUV (open triangles), DUD (open circles), and ChEMBL (filled circles). The thin line corresponds to $y = x$ while the linear regression curve is shown as a thick, black line.

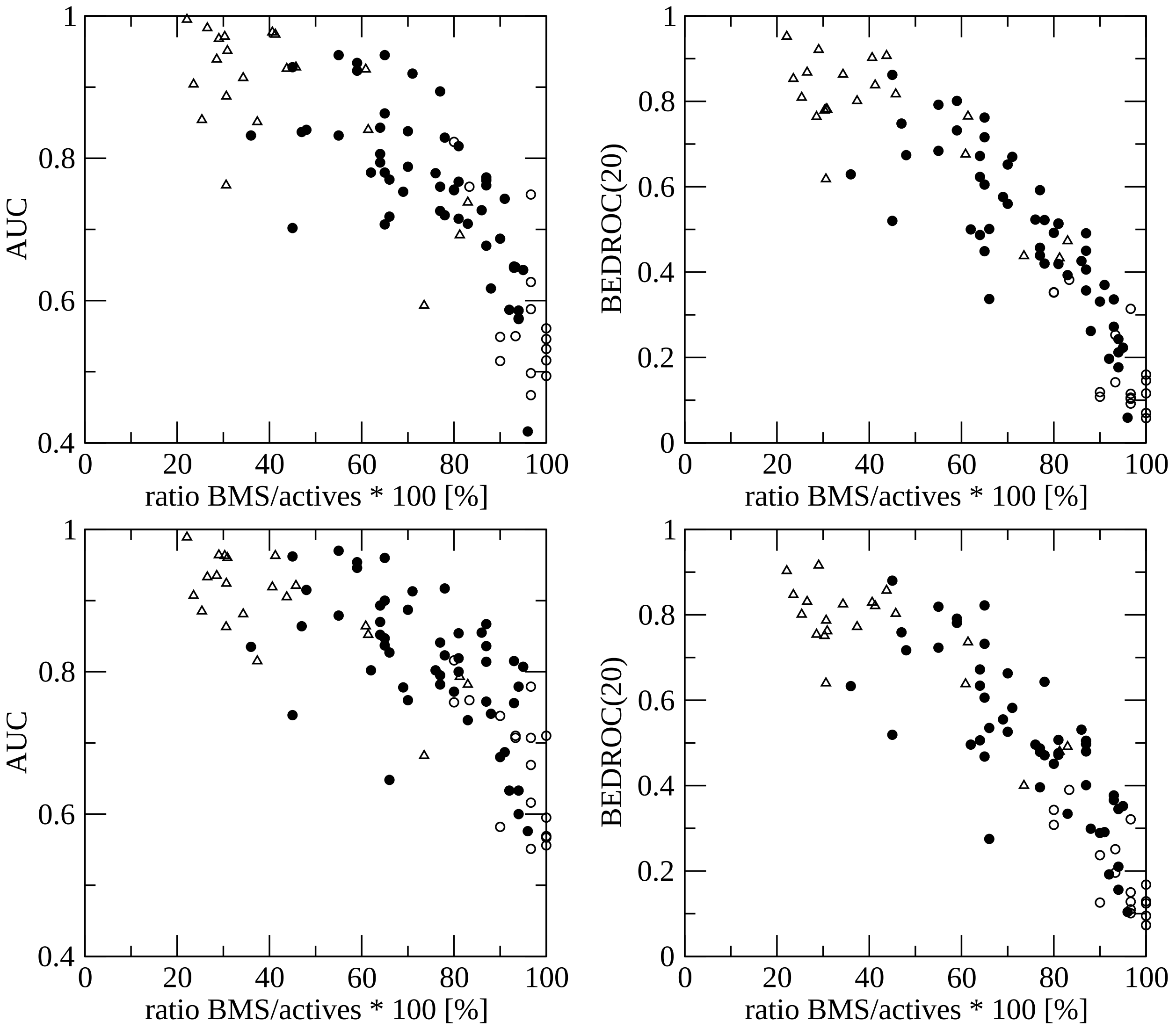

Figure S2: Average performance of ECFP4 (top panels) and TT (bottom panels) determined by AUC (left) and BEDROC(20) (right) as a function of the ratio BMS/actives for the three collection of data sets: MUV (open triangles), DUD (open circles), and ChEMBL (filled circles).

Table S1: Slope A and constant B, correlation coefficient (r), coefficient of determination (r^2) and root-mean-square error (RMSE) of the linear regression curve of 12 correlations between evaluation methods for the fingerprint ECFP4. Values for all 88 targets and for a subset of 67 targets (MUV and ChEMBL) are given.

**Method 1**	**Method 2**	**Targets**	A	B	r	r^2	RMSE
EF(1%)	EF(5%)	88	0.221	2.595	0.823	0.678	2.740
		67	0.207	1.811	0.975	0.950	0.936
BEDROC(20)	BEDROC(100)	88	1.131	-0.063	0.986	0.971	0.049
		67	1.140	-0.072	0.987	0.975	0.040
RIE(20)	RIE(100)	88	2.977	0.385	0.927	0.860	4.808
		67	3.660	-2.915	0.990	0.980	1.950
BEDROC(20)	RIE(20)	88	15.745	0.714	0.989	0.977	0.601
		67	17.528	0.203	0.999	0.998	0.152
BEDROC(20)	EF(1%)	88	58.832	3.094	0.820	0.672	10.312
		67	89.954	-6.335	0.983	0.965	3.662
BEDROC(20)	EF(5%)	88	19.183	0.199	0.998	0.995	0.334
		67	19.415	0.175	0.998	0.996	0.257
RIE(100)	EF(1%)	88	1.389	-3.515	0.990	0.980	2.528
		67	1.403	-3.316	0.994	0.988	2.169
RIE(20)	EF(5%)	88	1.197	-0.487	0.991	0.983	0.636
		67	1.108	-0.049	0.999	0.998	0.204
AUC	BEDROC(20)	88	1.670	-0.770	0.946	0.895	0.081
		67	1.573	-0.718	0.943	0.889	0.072
AUC	RIE(20)	88	26.660	-11.686	0.949	0.900	1.263
		67	27.751	-12.512	0.948	0.898	1.204
AUC	EF(1%)	88	97.316	-41.495	0.769	0.591	11.522
		67	135.882	-66.890	0.889	0.791	9.011
AUC	EF(5%)	88	32.352	-14.810	0.954	0.909	1.454
		67	30.832	-13.976	0.950	0.902	1.311

Table S2: Slope A and constant B, correlation coefficient (r), coefficient of determination (r^2) and root-mean-square error (RMSE) of the linear regression curve of 24 correlations between fingerprints (FP) for the evaluation method BEDROC(20). Linear regression was performed over all 88 targets.

**FP 1**	**FP 2**	A	B	r	r^2	RMSE
ECFC0	MACCS	1.054	0.0257	0.941	0.885	0.080
ECFC0	ECFC4	1.121	0.1026	0.948	0.899	0.079
MACCS	AP	0.953	0.1019	0.944	0.891	0.079
MACCS	ECFP4	1.006	0.1020	0.948	0.898	0.080
MACCS	FCFC4	1.001	0.0688	0.949	0.901	0.079
MACCS	Avalon	0.968	0.0966	0.952	0.906	0.074
AP	TT	0.987	0.0330	0.982	0.965	0.045
AP	ECFP4	1.024	0.0094	0.974	0.948	0.057
AP	Avalon	0.966	0.0170	0.958	0.918	0.069
AP	RDK5	0.966	0.0209	0.964	0.929	0.064
AP	FCFC4	1.020	-0.0240	0.977	0.954	0.053
AP	ECFC4	1.027	0.0038	0.983	0.965	0.046
Avalon	long Avalon	1.008	-0.0070	0.996	0.993	0.020
Avalon	RDK5	0.971	0.0178	0.977	0.954	0.051
Avalon	ECFP4	1.001	0.0196	0.960	0.921	0.070
ECFP4	TT	0.940	0.0358	0.984	0.968	0.043
ECFP4	long ECFP4	1.009	0.0023	0.999	0.998	0.012
ECFP4	ECFC4	0.985	0.0030	0.991	0.983	0.033
ECFP4	ECFP6	0.993	-0.0001	0.999	0.999	0.010
ECFP4	FCFP4	0.993	-0.0065	0.986	0.972	0.042
ECFP4	FCFC4	0.978	-0.0241	0.984	0.968	0.044
ECFP6	long ECFP6	1.012	0.0022	0.998	0.996	0.015
FCFP4	FCFC4	0.973	-0.0120	0.986	0.973	0.041
ECFC4	FCFC4	0.988	-0.0250	0.989	0.977	0.038

Table S3: Average rank of the 14 2D fingerprints averaged over 50 repetitions and 88 targets.

**Fingerprint**	**AUC**	**EF**		**BEDROC**		**RIE**	
		1 %	5 %	20	100	20	100
ECFC0	8.7±3.4	8.0±3.7	8.7±3.4	8.7±3.1	8.5±3.6	8.5±3.6	8.5±3.6
MACCS	7.4±3.9	6.5±4.2	7.4±3.9	6.2±4.0	6.6±4.1	7.1±4.1	7.1±4.1
AP	8.4±4.0	6.0±4.1	8.4±4.0	9.4±4.0	7.8±4.2	7.4±4.2	7.4±4.2
TT	6.1±3.5	5.1±3.7	6.1±3.5	4.8±3.5	5.2±3.5	5.7±3.6	5.7±3.6
Avalon	7.9±3.7	6.5±4.2	7.9±3.7	9.2±3.6	8.1±4.1	7.5±4.0	7.5±4.0
lAvalon	5.8±3.0	8.3±3.4	5.8±3.0	6.3±2.9	7.0±3.1	6.7±3.1	6.7±3.1
RDK5	11.7±3.0	10.0±3.8	11.7±3.0	10.7±3.8	10.5±3.8	11.3±3.4	11.3±3.4
ECFP4	5.5±2.7	8.1±3.3	5.5±2.7	6.6±2.9	7.1±3.1	6.3±2.9	6.3±2.9
lECFP4	4.6±2.7	7.5±3.6	4.6±2.7	4.4±2.4	5.5±2.9	5.5±3.0	5.5±3.0
ECFP6	4.9±2.9	8.0±3.9	4.9±2.9	4.1±2.5	5.3±3.1	5.8±3.3	5.8±3.3
lECFP6	7.6±3.5	7.1±3.8	7.6±3.5	7.2±3.3	7.4±3.7	7.6±3.7	7.6±3.7
FCFP4	13.0±2.5	10.1±4.0	13.0±2.5	13.1±2.2	12.2±3.1	12.4±3.1	12.4±3.1
ECFC4	6.6±3.6	7.5±4.1	6.6±3.6	6.7±3.3	6.8±3.8	6.8±3.9	6.8±3.9
FCFC4	6.6±3.1	6.4±3.2	6.6±3.1	7.7±3.2	7.1±3.4	6.4±3.2	6.4±3.2

Table S4: Results from pairwise post-hoc Friedman tests of the average rank between 14 2D fingerprints for the evaluation methods EF(1%) (top) and EF(5%) (bottom). Pairs of fingerprints with no statistically significant difference are marked with "X", pairs with an adjusted p-value distribution around the confidence level α with "o", and pairs with a statistically significant difference with "-". Fingerprints are ordered according to ascending average rank.

	**lECFP6**	**lECFP4**	**TT**	**RDK5**	**ECFP6**	**ECFP4**	**FCFP4**	**lAvalon**	**ECFC4**	**FCFC4**	**Avalon**	**AP**	**MACCS**	**ECFC0**	**Rank**
**lECFP6**		X	X	-	-	-	-	-	-	-	-	-	-	-	1
**lECFP4**			X	-	-	-	-	-	-	-	-	-	-	-	1
**TT**				o	o	-	-	-	-	-	-	-	-	-	1
**RDK5**					X	X	X	X	o	-	-	-	-	-	4
**ECFP6**						X	X	X	o	-	-	-	-	-	4
**ECFP4**							X	X	X	-	-	-	-	-	4
**FCFP4**								X	X	-	-	-	-	-	4
**lAvalon**									X	o	-	-	-	-	4
**ECFC4**										X	X	o	-	-	4
**FCFC4**											X	X	X	-	4
**Avalon**												X	X	-	4
**AP**													X	-	4
**MACCS**														o	4
**ECFC0**															14

	**TT**	**lECFP6**	**lECFP4**	**RDK5**	**FCFP4**	**ECFP6**	**ECFP4**	**ECFC4**	**lAvalon**	**AP**	**Avalon**	**FCFC4**	**MACCS**	**ECFC0**	**Rank**
**TT**		X	X	o	-	-	-	-	-	-	-	-	-	-	1
**lECFP6**			X	o	o	-	-	-	-	-	-	-	-	-	1
**lECFP4**				X	o	-	-	-	-	-	-	-	-	-	1
**RDK5**					X	X	X	X	X	X	-	-	-	-	1
**FCFP4**						X	X	X	X	X	o	-	-	-	1
**ECFP6**							X	X	X	X	X	o	-	-	1
**ECFP4**								X	X	X	X	X	-	-	1
**ECFC4**									X	X	X	o	-	-	1
**lAvalon**										X	X	X	-	-	1
**AP**											X	X	-	-	1
**Avalon**												X	-	-	1
**FCFC4**													o	-	1
**MACCS**														X	13
**ECFC0**															13

Table S5: Results from pairwise post-hoc Friedman tests of the average rank between 14 2D fingerprints for the evaluation method BEDROC(100). Pairs of fingerprints with no statistically significant difference are marked with "X", pairs with an adjusted p-value distribution around the confidence level α with "o", and pairs with a statistically significant difference with "-". Fingerprints are ordered according to ascending average rank.

	**lECFP4**	**lECFP6**	**ECFP4**	**ECFP6**	**TT**	**FCFP4**	**ECFC4**	**RDK5**	**lAvalon**	**Avalon**	**AP**	**FCFC4**	**MACCS**	**ECFC0**	**Rank**
**lECFP4**		X	X	o	o	-	-	-	-	-	-	-	-	-	1
**lECFP6**			X	X	X	-	-	-	-	-	-	-	-	-	1
**ECFP4**				X	X	X	X	-	-	-	-	-	-	-	1
**ECFP6**					X	X	X	o	o	-	-	-	-	-	1
**TT**						X	X	o	o	o	-	-	-	-	1
**FCFP4**							X	X	X	X	o	-	-	-	1
**ECFC4**								X	X	X	o	-	-	-	1
**RDK5**									X	X	X	o	-	-	1
**lAvalon**										X	X	o	-	-	1
**Avalon**											X	X	-	-	1
**AP**												X	-	-	1
**FCFC4**													-	-	1
**MACCS**														X	13
**ECFC0**															13
